# Treatment planning methodology for the Miami Multichannel Applicator following the American Brachytherapy Society recently published guidelines: the Lahey Clinic experience

**DOI:** 10.1120/jacmp.v14i1.4098

**Published:** 2013-01-07

**Authors:** Ileana Iftimia, Eileen T. Cirino, Herbert W. Mower, Andrea B. McKee

**Affiliations:** ^1^ Radiation Oncology Department Lahey Clinic Burlington MA; ^2^ Tufts University School of Medicine Boston MA USA

**Keywords:** GYN HDR brachytherapy, Miami Multichannel Applicator, standardized procedure, ABS guidelines

## Abstract

The objective of this study was to develop a standardized procedure from simulation to treatment delivery for the multichannel Miami applicator, in order to increase planning consistency and reduce errors. A plan is generated prior to the 1st treatment using the CT images acquired with the applicator in place, and used for all 3 fractions. To confirm the application placement before each treatment fraction, an AP image is acquired and compared with the AP baseline image taken at simulation. A preplanning table is generated using the EBRT doses and is used to compute the maximum allowable D2cc for bladder, rectum, and sigmoid, and the mean allowable dose for the upper vaginal wall per HDR brachytherapy fraction. These data are used to establish the criteria for treatment planning dose optimization. A step‐by‐step treatment planning approach was developed to ensure appropriate coverage for the tumor (D90>100% prescribed dose of 700 cGy/fraction) and the uninvolved vaginal surface (dose for the entire treatment length > 600 cGy/fraction), while keeping the organs at risk below the tolerance doses. The equivalent dose 2 Gy (EQD2) tolerances for the critical structures are based on the American Brachytherapy Society (ABS) recently published guidelines. An independent second check is performed before the 1st treatment using an in‐house Excel spreadsheet. This methodology was successfully applied for our first few cases. For these patients: the cumulative tumor dose was 74–79 EQD2 Gy10 (ABS recommended range 70–85); tumor D90 was >100% of prescribed dose (range 101%–105%); cumulative D2cc for bladder, rectum, and sigmoid were lower than the tolerances of 90, 75, and 75 EQD2 Gy3, respectively; cumulative upper vaginal wall mean dose was below the tolerance of 120 EQD2 Gy3; the second check agreement was within 5%. By using a standardized procedure the planning consistency was increased and all dosimetric criteria were met.

PACS numbers: 87.55‐x, 87.56 bg

## I. INTRODUCTION

Vaginal cuff brachytherapy is performed either alone or in combination with external beam radiation therapy (EBRT) for patients with endometrial or cervical carcinomas.^(^ ^1^ ^,^ ^2^ ^)^ Most authors agree that the upper third of the vagina should be the target for this group of patients because the majority of the treatment failures were seen in this region.^(^ ^3^ ^–^ ^4^ ^)^ There is still no consensus regarding applicator type but, in general, the high dose rate (HDR) brachytherapy is performed in multiple fractions using a vaginal cylinder.^(^ ^5^ ^)^

A single‐channel vaginal cylinder is most commonly used to prevent the recurrence of endometrial cancer in the vagina, as an adjuvant therapy after a hysterectomy, and also to treat very early (< 5 mm) vaginal carcinomas. However, with a single central channel, the possibilities for adjustment of the dose distribution are limited and may not always provide optimal distribution of the target dose while keeping the critical structures below the tolerance levels.

Interstitial brachytherapy is the treatment of choice for deeper lesions which extend beyond 5 mm (e.g., Syed applicator).^(^ ^6^ ^)^ To avoid using such an invasive approach, a multichannel vaginal cylinder may be the applicator of choice for small tumors extending up to 1 cm depth. By using such a device, an asymmetric dose distribution can be achieved, such that the vaginal mucosa dose is kept low in all areas except for a small region where the tumor is located. Also, the dose to critical structures may be reduced.^(^ ^7^ ^,^ ^8^ ^)^

Recently we purchased a Miami 7‐channel kit (Mick Radio‐Nuclear Instruments, Inc., CT Compatible Miami GYN Applicator for Varian GammaMed) and implemented the Miami HDR brachytherapy approach in our clinic.

For HDR brachytherapy, it is extremely important to efficiently perform all tasks, from simulation to treatment delivery, and to do a second check prior to treatment. There is currently little guidance about the treatment planning methodology for the Miami applicator and there is limited published literature on this topic.^(^ ^7^ ^,^ ^10^ ^)^ Our group developed a standardized procedure in order to achieve an appropriate plan in a timely manner, to reduce the risk of errors in treatment delivery and to increase patient safety. This methodology is presented here, along with the preliminary results obtained. This experience may prove useful for other centers.

## II. MATERIALS AND METHODS

For the single‐channel vaginal cylinder cases, CT‐based treatment planning is performed and the dose for the bladder and rectum is recorded. With this simple device, the plan cannot be optimized to reduce the dose to critical structures without compromising the prescription dose. The Miami multichannel cylinder is more versatile from this point of view. It offers a means to optimize the dose based on the patient anatomy. An asymmetric dose distribution along the axis of the cylinder can be generated and the dose to critical structures may be reduced. For treatment planning consistency regarding tumor dose and tolerance doses for the critical structures, we adopted the American Brachytherapy Society (ABS) recently published consensus guidelines for interstitial brachytherapy for vaginal cancer,^(^ ^11^ ^)^ as described in the following.

**Figure 1:**
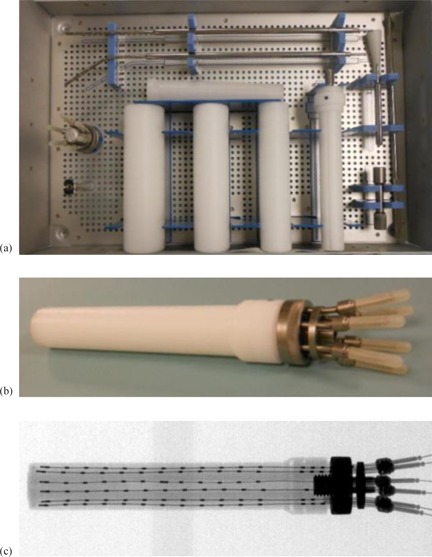
The Miami kit (a) in the sterilization box; photo of the Miami cylinder (b) with a 25 mm buildup cap; digital radiographic image (c) of the Miami cylinder with a 25 mm buildup cap and dummy wires inserted in all 6 peripheral channels.

Our Miami kit contains a cylindrical obturator with 6 peripheral channels, and a central channel which can accommodate a straight or a curved tandem that can be used to treat the disease in the cervical uterus. It also includes four buildup caps (25, 30, 35, and 40 mm diameter). A photo of the Miami kit in the sterilization box is shown in Fig. 1(a). Figures 1(b) and (c) show a photo and a radiograph, respectively, of the multichannel cylinder with a 25 mm buildup cap. Dummy wires were inserted in all 6 peripheral channels before the digital radiographic image was acquired.

Most patients treated using the Miami approach have 25 to 28 fractions EBRT prior to the HDR brachytherapy boost. The radiation oncologist will implant fiducial markers around the tumor prior to the CT simulation for the HDR brachytherapy boost. This will assist in the tumor contouring process. Alternatively, MR images can be acquired with an MR compatible single‐channel vaginal cylinder in place, with a diameter equal to that of the Miami multichannel cylinder to be used. (The Miami multichannel cylinder kit purchased is not MR‐compatible. The manufacturer is currently testing MR compatibility.)

In addition, a detailed analysis of the EBRT treatment plan is done prior to the CT simulation for the HDR brachytherapy treatment. The following information is obtained: EBRT prescription dose and number of fractions; the D2cc for bladder, rectum, sigmoid; and D2cc, D1cc, and mean dose for the upper vagina. This information is entered in a table (see Table 1 as an example), designed to estimate the maximum D2cc the critical structures can receive from the HDR brachytherapy boost procedure. All tolerances in this table are given in terms of equivalent dose 2 Gy (EQD2).^(^ ^11^ ^)^

**Table 1:** EQD2‐BED preplanning table. In this example the HDR brachytherapy D2cc should be lower than 6.9, 5.2, and 5.2 Gy/fraction for the bladder, rectum, and sigmoid, respectively, to keep the cumulative dose below the tolerance value for those structures. Also, the upper vaginal mean dose should be limited to 9.5 Gy/fraction. These values are used during planning as constraints for the dose to critical structures.

Patient name:_______________			ID #:___________				
*Input Data*							
	*Tumor (PTV1)*	*Bladder D2cc*	*Rectum D2cc*	*Sigmoid D2cc*	*Vaginal Mean dose*	*Upper Vagina D2cc*	*Upper Vagina D1cc*
EBRT Total Dose [Gy] (25 Fx)	45	49	49	49	49	49	49
HDR Dose/Fx [Gy] (3 Fx)	7.0	6.9	5.2	5.2	9.5	9.5	9.5
*Output Data BED/EQD2*							
BED EBRT	53.1	81.0	81.0	81.0	81.0	81.0	81.0
BED HDR	35.7	68.3	42.6	42.6	118.5	118.5	118.5
Total BED	88.8	149.3	123.7	123.7	199.5	199.5	199.5
BED tol.		150	125	125	200	200	200
Total EQD2	74.0	89.6	74.2	74.2	119.7	119.7	119.7
EQD2 Gy3 tol.		90	75	75	120	120	120
EQD2 Gy10 range	70–85						
Physicist:__________________			Date:_______________				

Notes: All doses are in Gy; Alpha/beta ratio 3 Gy for OAR (late effects), 10 Gy for tumor; Tolerances based on ABS/GEC‐ESTRO guidelines for GYN.

The cumulative biologically equivalent dose (BED) is given by the sum of BED for the EBRT and HDR brachytherapy procedures (Eq. (1)). The BED and EQD2 can be calculated using Eq. (2), where *n* is the number of fractions, *d* is the dose per fraction, and alpha/beta (α/β) ratio is considered 10 Gy for the tumor and 3 Gy for the critical structures (late effects). EQD2 Gy10, and EQD2 Gy3 are the notations for the EQD2 when the α/β ratio is 10 and 3 Gy, respectively.

(1) CUM BED = BED(EBRT)+BED(HDR)

(2) $$\text{With}\{\begin{array}{l} \text{BED}=\text{nd}\left(1+\frac{d}{\alpha {/}_{\beta }}\right)\\ \text{EQD}2=\frac{\text{BED}}{1+\frac{2}{\alpha {/}_{\beta }}} \end{array}$$

In the example shown in Table 1, the HDR brachytherapy D2cc should be lower than 6.9, 5.2, and 5.2 Gy/fraction for the bladder, rectum, and sigmoid, respectively, to keep the cumulative dose below the tolerance value for those structures. Also, the upper vaginal mean dose should be limited to 9.5 Gy/fraction. During treatment planning these values are used as constraints for the dose to critical structures.

A single treatment plan is currently generated for all 3 HDR brachytherapy fractions, using the CT images acquired with the applicator in place before the 1st HDR brachytherapy treatment.

**Figure 2:**
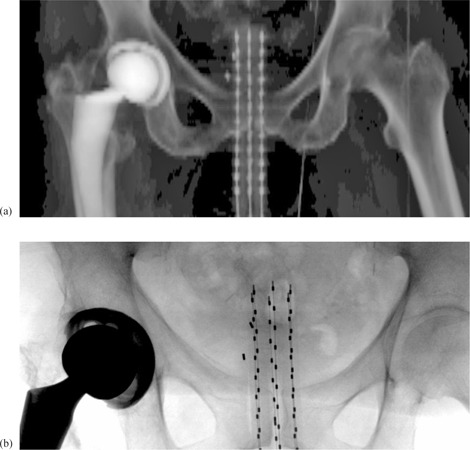
Example of an AP CT scout image (a) used as a baseline; example of an AP digital radiographic image (b) taken before a treatment fraction. Coded dummy wires are placed inside each peripheral channel, as shown.

The CT simulation for the HDR brachytherapy procedure is performed as follows. The Miami applicator is assembled by using an appropriate buildup cap. Dummy wires are placed inside all peripheral channels and then the applicator is inserted into the patient. In order to maintain the device at a consistent position for simulation and treatments, the cylinder is rotated to orient the center of channel 4 anteriorly, and then kept in that position by using a Universal applicator clamping device (Varian Medical Systems, Palo Alto, CA). Unlike when a single‐channel cylinder is used, the rotation of the patient and the relative position of the device in the patient are important in the case of a Miami device. Since most patients treated using the Miami approach have EBRT delivered prior to the HDR brachytherapy, they have tattoos for the EBRT isocenter. To maintain the same position for both HDR brachytherapy simulation and treatments, the CT lasers are aligned with the EBRT tattoos. For patients not having EBRT treatments, the tattooing is done during the CT simulation for the HDR brachytherapy. CT images (3 mm slice thickness) are then acquired and exported to the Varian BrachyVision 10 treatment planning system (TPS) (Varian Medical Systems). After simulation, an AP scout image (see Fig. 2(a) is printed and used as a baseline for the pretreatment verification procedure.

CT‐based treatment planning using the Varian BrachyVision 10 TPS is performed following the steps described below. CT images are imported and then the radiation oncologist contours the tumor (labeled PTV1 in the following) and establishes the treatment length, guided by the fiducial markers. The critical structures are contoured by a physicist and verified by the radiation oncologist. If MR images are acquired, the MR and CT images are imported and coregistered and then the PTV1 and critical structures (bladder, rectum, and sigmoid) are contoured on the MR images. The applicator buildup cap is contoured on the CT images and then a structure labeled PTV2, defined as the applicator buildup cap, is extended inferiorly for the entire treatment length. The lateral surface of PTV2, excluding the part overlapped with PTV1, is referred in the following as the uninvolved vaginal surface. The upper vaginal wall is defined as a 1 mm thick lateral cylindrical shell around the PTV2, so this includes the involved and uninvolved vaginal wall. A new structure labeled “buildup cap+1 cm” is defined by adding 1 cm margins to the buildup cap. If the PTV1 extends outside the “buildup cap+1 cm” structure, the radiation oncologist analyzes the case and decides if the Miami approach is indeed appropriate. After all contours are drawn, the 6 peripheral channels are defined using the dummy wires as guides. These 6 channels are engraved with numbers from 2 to 7, clockwise, with channel 4 located anteriorly. Channel number 1 is reserved for a tandem. If a buildup cap with a diameter 30, 35, or 40 mm is used, in BrachyVision 10 TPS the applicator can be loaded from the applicators library. The peripheral channels are then loaded up to the treatment length with equal dwell times for all dwell positions (e.g., 3 sec).

For this HDR brachytherapy approach, we prescribe 2100 cGy in 3 fractions. The prescription is written as follows: tumor (PTV1) D90>100% of prescribed dose (700 cGy/fraction); the dose to the uninvolved vaginal surface at least 600 cGy/fraction; 5 mm step size; 1 fraction/day, 1–2 fractions/week; treatment length (usually 5 cm); and the buildup cap diameter as defined at simulation. Other dose schemes could conceivably be used, per radiation oncologist's decision.

The plan is generated by using volume‐based optimization. Constraints are set for the PTV1 and PTV2 to be covered with 700 cGy/fraction and 600 cGy/fraction, respectively, and also for the maximum bladder, rectum, and sigmoid doses. The constraints for the critical structures are established based on the maximum allowable HDR brachytherapy D2cc values for the patient (taken from a table similar to Table 1, prepared before planning). To increase the computation accuracy, the resolution is adjusted for all structures as needed (> 5000 points preferable). After the plan is optimized, a manual shaping feature can be used to adjust the dose locally in order to meet all dosimetric criteria (i.e., the upper vaginal wall mean dose to become less than the tolerance value, the cumulative D2cc for bladder, rectum, and sigmoid to remain below the corresponding tolerance levels, while the PTV1 and PTV2 to be properly covered with the prescribed doses). Depending on tumor volume, extent, and location, if the tumor covers a relatively large area of the vaginal surface, the upper vaginal wall D1cc may exceed the tolerance value but the D2cc would still be below the tolerance. When all cumulative dose‐volume histogram (DVH) criteria are met, a new table is filled out with the cumulative (EBRT plus HDR brachytherapy) EQD2‐BED dose for the tumor (PTV1), D2cc for bladder, rectum, sigmoid, D1cc, D2cc, and mean dose for upper vaginal wall (example given in the Results and Discussion section below). Furthermore, D1cc and D0.1cc are listed in the plan report for bladder, rectum, and sigmoid, along with PTV1 V100, V150, and V200 used to evaluate target coverage and hot spots. In addition, dose recording points are defined as recommended by the ABS guidelines:^(^ ^12^ ^)^ right and left side, on the buildup cap surface and at 5 mm from the surface, at 1.25 cm inferior from the buildup cap distal end. The dose to these points is also listed in the plan report.

A reference line with multiple points is defined at the buildup cap surface, on the contralateral side relative to the tumor position. This is used to record the dose to the uninvolved vaginal surface. Multiple calculation points are generated as well, to be used for second check. This second check is performed by another physicist prior to the 1st treatment using an in‐house Excel spreadsheet template based on the point source approximation approach.^(^ ^13^ ^)^ The person performing the second check completes a checklist (see Appendix 1). The plan report, DVH plot, isodose distribution, second check file, checklist for the second check, and the EQD2‐BED postplanning table are kept in the patient electronic record. To help the planning process, templates were created in BrachyVision 10 TPS for structure names, plan report, isodose levels to be displayed, and dose optimization criteria.

Before each HDR brachytherapy fraction, the patient is placed on the Varian Acuity conventional simulator couch^(^ ^14^ ^)^ for a pretreatment verification, to assess if the applicator is in the same location as it was during the CT simulation. The Miami applicator with the buildup cap used for simulation and dummy wires placed in all peripheral channels is inserted into the patient. The device is rotated with channel 4 anteriorly, and then kept in that position by using a universal applicator clamping device. The conventional simulator lasers are aligned with the EBRT tattoos, as it was done during the HDR brachytherapy simulation. An AP digital radiographic image (see Fig. 2(b) is taken by using the conventional simulator and is compared with the baseline AP CT scout image shown in Fig. 2(a). To evaluate the applicator position, the dummy dots above the pelvic rim are counted on the image. Additionally, the position of the distal end of the channels relative to the tumor fiducial markers can be used for this assessment. The patient can then be treated on the couch in the same position as during verification, since the HDR brachytherapy unit (Varian GammaMed, Varian Medical Systems) is located in the same room with the conventional simulator.

Checklists were designed for the therapists and physicists for simulation and treatments. Two important items included in these checklists to be verified are: (1) the diameter of the buildup cap, and (2) the device rotational position (channel 4 anterior). The Physics–HDR Brachytherapy Treatment Delivery Checklist is shown in Appendix 2 as an example.

## III. RESULTS & DISCUSSION

The methodology described above was successfully applied for our initial cases. All patients received either 4500 cGy (in 25 fractions) or 5040 cGy (in 28 fractions) from the EBRT treatment delivered before the HDR brachytherapy boost. A single treatment plan was generated for all 3 HDR brachytherapy fractions, as mentioned above. Future work will be done to evaluate if there is a clinically significant benefit when CT images are acquired and a treatment plan is generated for each fraction. Symon et al.^(^ ^10^ ^)^ performed such an analysis and concluded that individual fraction optimization may be important to minimize doses to critical structures.

To date we have used the Miami system without a central tandem. For future cases, we will investigate the use of the central channel for treatment of vaginal tumors. This may help the dose shaping and critical structure sparing. Based on our preliminary tests, the use of a central channel may allow for a more homogeneous dose distribution on the surface of the applicator, thus reducing the D1cc and D2cc for the uninvolved vaginal wall.

For all patients studied here, the cumulative (EBRT plus HDR) tumor (PTV1) dose was 74–79 EQD2 Gy10 (ABS recommended range for GYN cases is 70–85 EQD2 Gy10), and the cumulative D2cc for bladder, rectum, and sigmoid were lower than the tolerances of 90, 75, and 75 EQD2 Gy3, respectively.^(^ ^11^ ^)^ For all cases, we were able to achieve the goal of tumor (PTV1) D90>100% of the prescribed dose (range 101%–105% of prescribed dose), and at the same time keep the cumulative upper vaginal wall mean dose below the tolerance of 120 EQD2 Gy3 (corresponding BED 200). PTV1 DVH parameters V100, V150, and V200 were >90%, <50%, and <30%, respectively. The discrepancy between the calculation point dose estimated with the BrachyVision TPS and using the in‐house Excel spreadsheet was less than 5% for all patients.

Table 2 shows an example of EQD2‐BED doses recorded after planning. The PTV1 was covered with a cumulative dose of EQD2 Gy10=79.3, within the range recommended by the ABS guidelines. The cumulative EQD2‐BED was below the tolerance dose for the bladder, rectum, and sigmoid. The cumulative upper vaginal wall mean dose was limited to the tolerance level. Also, cumulative D2cc and D1cc for the upper vaginal wall were below the tolerance values for this case.

**Table 2:** EQD2‐BED postplanning table. The tumor (PTV1) was covered with a cumulative dose of EQD2 Gy10=79.3, located in the range recommended by the ABS guidelines. The cumulative EQD2‐BED is below the tolerance dose for the bladder, rectum, and sigmoid. The cumulative upper vaginal mean dose is limited to the tolerance level. Also, cumulative D2cc and D1cc for the upper vagina are below the tolerance values.

Patient name:_______________			ID #:_____________				
*Input Data*							
	*Tumor (PTV1)*	*Bladder D2cc*	*Rectum D2cc*	*Sigmoid D2cc*	*Upper Vaginal Mean dose*	*Upper Vagina D2cc*	*Upper Vagina D1cc*
EBRT Total Dose [Gy] (28 Fx)	50.4	51.7	51.7	51.6	51.4	51.4	51.4
HDR Dose/Fx [Gy] (3 Fx)	7.0	2.4	4.1	4.1	9.4	5.7	7.4
*Output Data BED/EQD2*							
BED EBRT	59.5	83.5	83.5	83.3	82.9	82.9	82.9
BED HDR	35.7	13.1	28.9	29.0	116.6	49.2	76.4
Total BED	95.2	96.6	112.4	112.3	199.4	132.0	159.3
BED tol.		150	125	125	200	200	200
Total EQD2	79.3	58.0	67.4	67.4	119.7	79.2	95.6
EQD2 Gy3 tol.		90	75	75	120	120	120
EQD2 Gy10 range	70–85						
Physicist:__________________			Date:_______________				

Notes: All doses are in Gy; Alpha/beta ratio 3 Gy for OAR (late effects), 10 Gy for tumor; Tolerances based on ABS/GEC‐ESTRO guidelines for GYN.

**Figure 3:**
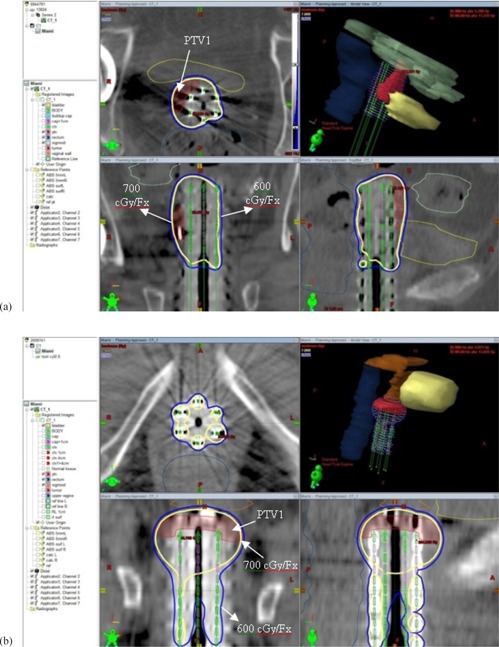
Examples of Miami treatment plan dose distribution: (a) tumor unilaterally located – patient right side; (b) tumor superiorly located, almost symmetric around the vaginal cuff. The critical structures bladder, rectum, and sigmoid are contoured, as shown. The prescribed dose of 700 cGy/fraction covers the tumor (PTV1) while the uninvolved vaginal surface is covered with 600 cGy/fraction.

Figures 3(a) and (b) show examples of dose distribution for two cases: a) tumor laterally located; b) tumor superiorly located. The situation presented in Fig. 3(a) is for a case when the tumor is unilaterally located, so the Miami approach could achieve very good results by generating an asymmetric dose distribution. In the example presented in Fig. 3(b) the tumor is superiorly located, almost symmetric around the vaginal cuff. The main concern here was the sigmoid, which was located very close to the tumor. By using the Miami applicator we were able to meet all criteria and keep the critical structures below the tolerance doses.

**Figure 4:**
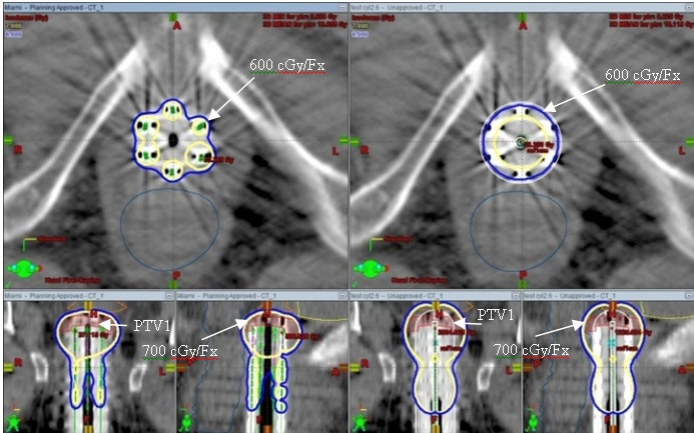
Isodose line distributions for two plans are displayed for comparison. Left side: treatment plan was obtained by using a Miami multichannel vaginal cylinder. Right side: treatment plan was obtained by using a single‐channel vaginal cylinder; dose distribution was generated by using a differential normalization. For both plans, the prescribed dose of 700 cGy/fraction covers the tumor (PTV1), while the uninvolved vaginal surface is covered with 600 cGy/fraction. PTV1 D90 was comparable for both plans (slightly >100% of prescribed dose).

**Figure 5:**
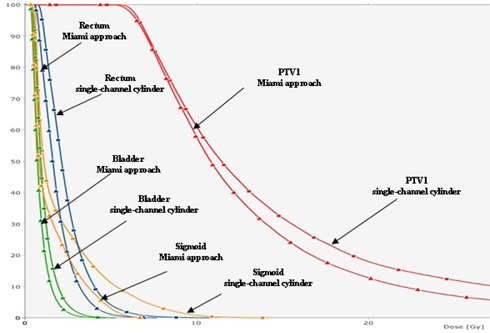
DVH comparison for two plans: using a Miami multichannel vaginal cylinder (continuous lines with triangle symbols) and a single‐channel vaginal cylinder (continuous lines with square symbols). The dose to critical structures (bladder, rectum, sigmoid) was lower for the Miami approach. The D2cc for the sigmoid was above the tolerance value for the single‐channel vaginal cylinder. It was somewhat easier to keep the upper vaginal wall mean dose below the tolerance value with a single‐channel vaginal cylinder, though the D1cc and D2cc were higher than the values obtained when using the Miami applicator. Also, the tumor (PTV1) V150 and V200 are higher when using a single‐channel vaginal cylinder.

Because the example shown in Fig. 3(b) was a special case, we decided to perform a further investigation. Retrospectively we replanned it by using a single‐channel vaginal cylinder with the diameter almost identical to the Miami buildup cap used for this patient (2.6 cm diameter cylinder versus 2.5 cm Miami buildup cap). With the single‐channel cylinder, the desired dose distribution was generated by using a differential normalization (at the tumor surface in the upper part of the cylinder and at the cylinder surface for the rest of the vagina, up to the treatment length). The isodose line distributions for the two plans are displayed in Fig. 4 for comparison. PTV1 D90 was comparable for both plans (slightly >100% of prescribed dose), but the dose to critical structures was lower for the Miami approach (see the DVH comparison in Fig. 5). The D2cc for the sigmoid was below the tolerance dose for the Miami case. It was not possible to achieve this using the single‐channel vaginal cylinder, so the Miami approach was indeed a good choice for this case. With a single‐channel vaginal cylinder, it was somewhat easier to keep the upper vaginal wall mean dose below the tolerance value (which was 8.6 Gy/fraction when the single‐channel cylinder was used versus 9.4 Gy/fraction for the Miami applicator). The D1cc and D2cc for the upper vaginal wall were higher than the values obtained when using the Miami applicator. Also, the PTV1, V150, and V200 were somewhat higher when using a single‐channel vaginal cylinder. The authors concluded that, with the multichannel Miami applicator, the dose to the critical structures can be reduced at the expense of a slightly increased vaginal wall mean dose. These results are in agreement with published data.^(^ ^9^ ^)^

Since the D5cc for the walls of critical structures (bladder, rectum, and sigmoid) may be related to late toxicity,^(^ ^12^ ^)^ we decided to retrospectively contour these structure walls and record this parameter, which will also be monitored for all future patients. For the patients studied here, it was difficult to identify the critical structure walls on the CT images. The wall thickness is dependent on patient age and organ filling. Published literature^(^ ^15^ ^–^ ^17^ ^)^ shows that the bladder and rectal wall thickness is about 3–5 mm and the sigmoid wall thickness is about 1 mm. For consistency, the following structures were contoured for all patients: BW3mm (3 mm thick bladder wall), BW5mm (5 mm thick bladder wall), RW3mm (3 mm thick rectal wall), RW5mm (5 mm thick rectal wall), and SW1mm (1 mm thick sigmoid wall). The D5cc range for these structures for the group of patients studied here is given in Table 3. Patient follow‐up may prove if this parameter is a good indicator of late effects and toxicity.

**Table 3:** D5cc range for the critical structure walls for the group of patients studied. The following structure walls were contoured: BW3mm (3 mm thick bladder wall), BW5mm (5 mm thick bladder wall), RW3mm (3 mm thick rectal wall), RW5mm (5 mm thick rectal wall), and SW1mm (1 mm thick sigmoid wall).

	*BW3mm*	*BW5mm*	*RW3mm*	*RW5mm*	*SW1mm*
D5cc range [Gy]/Fx	1.4–3.0	1.7–3.6	2.6–2.8	3.0–3.2	0.7–1.7

## IV. CONCLUSIONS

Our preliminary results showed that a standardized procedure with this new applicator has the potential to improve plan quality and dosimetric consistency. It will be adopted by our staff and considered part of our standard practice for increasing patient safety. The information presented in this paper may help other physicists to more easily start a Miami HDR brachytherapy approach in their own clinics.

As discussed above, the applicator selection is very important and should be case‐based. In particular, for tumors unilaterally located or in close proximity to some critical structures, the Miami multichannel cylinder may be the applicator of choice. By using such a device, the dose to critical structures can be reduced, but this will result in a slight increase of the vaginal wall mean dose.

## Appendix 1. MIAMI GYn Applicator ‐ Checklist for Second Check

**Table:**

Patient Name:(Last, First, Middle Initial)	Therapy #: _____
Prescription verified (stepping distance, dose)	____	√
Applicator ID verified (2–7, ch. 4 anterior)	_____	√
Application direction verified ‐ arrow superior	_____	√
Total Dwell Time recorded	_____
Minimum Dwell Time reviewed (see Table)	_____	√
Points defined (ABS guidelines: cyl. surface & at 5 mm)	_____	√
Contours drawn (bladder, rectum, sigmoid, upper vagina)	_____	√
EQD2‐BED calculations performed	_____	√
$2^{\text{nd}}$ check calculation performed	_____	√
Accurate data transferred to GammaMed	_____	√
**DVH review:**
Tumor (PTV1)	D90	_____	>100%
HDR Rx 7 Gy/Fx, 3 Fx
EBRT 1.8 Gy/Fx, 25 Fx
Check completed by:		Date:	
GammaMedPlus Minimal Dwell ${\text{Times}}^{1}$
^1^CTM‐GM‐278B Varian Technical Bulletin 5/22/07 (see bulletin for full listing)

^*^ Nominal dwell time (BrachyVision plan) x decay factor ≥ recommended minimum time.

**Table:**

*Distance Between Dwells (mm)*	*# of Stops @ 5 mm/stop*	*Recommended Minimum Times (sec)* ^*^
5 ‐ 10	1 ‐ 2	0.2
15 ‐ 30	3 ‐ 6	0.3
35 ‐ 55	7 ‐ 11	0.4
60 ‐ 90	12 ‐ 18	0.5
95 ‐ 130	19 ‐ 26	0.6

## Appendix 2. Physics – Hdr Brachytherapy Treatment delivery Checklist

HDR Brachytherapy ‐ Treatment Delivery Checklist

**Table:**

1. Patient Name: _____	Date: _____
MR#: _____
	*MD*	*Physicist*
2. QA Performed		N/A		
3. Patient Consent				N/A
4. Prior to Treatment, MD to Perform Patient Identification				N/A
Patient gives name [required]				N/A
and Patient gives date of birth [Out Patients]				N/A
or Wrist band identification [In Patients]				N/A
5. Pretreatment Patient Survey		N/A		
Meter Model and S/N:				
Background Reading	mR/hr			
Patient Reading	mR/hr			
6. AP Image Reviewed				N/A
7. Miami Applicator Checks				
7.a Verify Buildup Cap Diameter		cm		or	
7.b Verify Setup (ch. 4 UP, holder screwed on the docking cage center of ch. 4 or 7)			or	
8. Prescription				N/A
9. Treatment Plan Done		N/A		
10. Independent Plan Verification		N/A		
11. Plan Approved by MD				N/A
12. Treatment Parameters on Display Checked		N/A		
13. “Time‐out:”			and	
Verify All Items 1–12 Done				
Verify on GammaMed Treatment PC: Patient Name, Fx #, and Total Dwell Time				
14. Physicist Present During Treatment		N/A		
15. MD Present During Treatment				N/A
16. Post Treatment Patient Survey		N/A		
Meter Model and S/N:				
Background Reading	mR/hr			
Patient Reading	mR/hr			
Area Reading	mR/hr			
17. MD Review of Treatment Summary				N/A
